# Relationship of organizational culture, teamwork and job satisfaction in interprofessional teams

**DOI:** 10.1186/s12913-015-0888-y

**Published:** 2015-06-23

**Authors:** Mirjam Körner, Markus A. Wirtz, Jürgen Bengel, Anja S. Göritz

**Affiliations:** Medical Psychology and Medical Sociology, University of Freiburg, Hebelstr. 29, 79104 Freiburg, Germany; Department of Research Methods, Institute of Psychology, University of Education Freiburg, Kunzenweg 21, 79117 Freiburg, Germany; Department of Rehabilitation Psychology and Psychotherapy, Institute of Psychology, University of Freiburg, Engelbergerstr. 41, 79085 Freiburg, Germany; Department of Occupational and Consumer Psychology, Institute of Psychology, University of Freiburg, Engelbergerstr. 41, 79085 Freiburg, Germany

**Keywords:** Organizational culture, Teamwork, Job satisfaction, Hospital, Health care

## Abstract

**Background:**

Team effectiveness is often explained on the basis of input-process-output (IPO) models. According to these models a relationship between organizational culture (input = I), interprofessional teamwork (process = P) and job satisfaction (output = O) is postulated. The aim of this study was to examine the relationship between these three aspects using structural analysis.

**Methods:**

A multi-center cross-sectional study with a survey of 272 employees was conducted in fifteen rehabilitation clinics with different indication fields in Germany. Structural equation modeling (SEM) was carried out using AMOS software version 20.0 (maximum-likelihood method).

**Results:**

Of 661 questionnaires sent out to members of the health care teams in the medical rehabilitation clinics, 275 were returned (41.6 %). Three questionnaires were excluded (missing data greater than 30 %), yielding a total of 272 employees that could be analyzed. The confirmatory models were supported by the data. The results showed that 35 % of job satisfaction is predicted by a structural equation model that includes both organizational culture and teamwork. The comparison of this predictive IPO model (*organizational culture (I), interprofessional teamwork (P), job satisfaction (O)*) and the predictive IO model (*organizational culture (I), job satisfaction (O)*) showed that the effect of organizational culture is completely mediated by interprofessional teamwork. The global fit indices are a little better for the IO model (TLI: .967, CFI: .972, RMSEA .052) than for the IPO model (TLI: .934, CFI: .943, RMSEA: .61), but the prediction of job satisfaction is better in the IPO model (R^2^ = 35 %) than in the IO model (R^2^ = 24 %).

**Conclusions:**

Our study results underpin the importance of interprofessional teamwork in health care organizations. To enhance interprofessional teamwork, team interventions can be recommended and should be supported. Further studies investigating the organizational culture and its impact on interprofessional teamwork and team effectiveness in health care are important.

**Electronic supplementary material:**

The online version of this article (doi:10.1186/s12913-015-0888-y) contains supplementary material, which is available to authorized users.

## Background

Patients with chronic diseases have complex health needs and typically require treatment by more than one health care discipline [1]. In Germany, chronic care is provided in in-patient facilities (“rehabilitation clinics”) with interprofessional teams [2]. These teams consist of professionals of at least two health care disciplines who work together toward a common goal to achieve an optimal outcome for their patients [3–6]. Usually, the teams comprise physicians, nurses, physiotherapists, sport therapists, psychotherapists, psychologists, social workers, and dieticians [6–8], with the particular team composition depending on the type of chronic diseases treated in the clinic.

Team effectiveness is often explained on the basis of input-process-output models (IPO) [7, 9–16]. IPO models describe the impact of input (e.g., organizational culture, team composition, structure of communication, task design) and the mediating process (e.g., communication, coordination, respect, conflict leadership) on team output (e.g., team performance, job satisfaction, well-being, cost effectiveness, quality of care, treatment outcome) [11, 12, 14]. IPO models are heterogeneous with regard to their complexity and the elements they include. To empirically examine an IPO model, researchers typically select several elements in the models [17–20].

Organizational characteristics, such as organizational culture, are important aspects for interprofessional teamwork, treatment quality and success [21, 22]. Studies have demonstrated that interprofessional teamwork is influenced by organizational culture [23, 24]. Further studies have shown that teamwork predicts job satisfaction [14, 25–28]. However, until now no study has investigated a single model which includes all of the constructs in one model. This means, in detail, organizational culture as input, interprofessional teamwork as process, and job satisfaction as output, as well as the mediating effect of interprofessional teamwork in health care.

### Organizational culture

Organizational culture is often considered as the precondition of teamwork in the organization. It is defined as the shared values, beliefs, or perceptions held by employees within an organization [29], and “is the social glue holding an organization together” ([30], p. 2). Schein [31] stated that organizational culture consists of the underlying assumptions and beliefs that the members of an organization share and that operate unconsciously. Mission, strategy, structure, leadership and human resource practices are important determinants of organizational culture [32]. An organization with a strong culture helps employees to accomplish their goals and tasks and be satisfied in their job [30]. Organizational culture is “an important explanatory variable for behavior and performance in the workplace” ([32], p. 116) and influences teamwork and treatment outcomes [32]. Moreover, organizational culture predicts job satisfaction [30, 33–35]. Existing studies focus on the impact of organizational culture on implementing interventions [36], quality improvement [37], patient safety [38], or performance [39, 40], or focus only on one professional group such as nurses (e.g. [41, 30]). Additionally, these studies were conducted in acute care centers or nursing homes. Only Strasser [32] performed a study in the interprofessional rehabilitation setting. They verified that team functioning differs significantly depending on the dominant organizational culture. The highest team functioning scores were achieved by teams with a more personal and dynamic organizational culture rather than those that were more bureaucratic and formal [32]. Organizational culture influences the implementation of interventions in health care organizations, therefore its characteristics need to be investigated in order to improve implementation processes, e.g. for guidelines [42]. There are only a few questionnaires for organizational culture which are clinic-specific (e.g. Hospital Culture Questionnaire [43], The Hospital Culture Scale [44]), or used in a clinical setting [45]. All of them have limitations concerning their psychometrical testing, structure and theoretical basis and vary in their definition of organizational culture [45].

### Interprofessional teamwork

Interprofessional teamwork is a key feature of the comprehensive chronic care approach [46–48]. It is defined as a partnership “in a participatory, collaborative and coordinated approach to shared decision-making around health and social issues” of the patients ([5], p. 11). Körner and Wirtz [49] define the core dimensions of client-centered interprofessional teamwork as communication, cooperation, coordination, respect, and work climate. Furthermore, teamwork in health care may be categorized into interprofessional versus multiprofessional team approaches [12, 28]. The two approaches differ in organization, leadership, communication and decision-making, with the interprofessional approach achieving better results in teamwork and higher staff satisfaction than the multiprofessional approach [28]. Multiprofessional teamwork means that the different disciplines/professions work separately, each with its own treatment goals. The physician determines and delegates the treatment options to the other health care professionals in a one-way, mostly bilateral interaction process between the professionals. In contrast, the interprofessional approach is more interactive and participative, with the health care professionals agreeing on a common treatment goal and adapting their discipline-specific goals to this common goal. The physician involves the other health care professionals in treatment decisions within a multilateral interaction process and coordinates the treatment in interprofessional team meetings [28, 50]. Several studies have shown the effects of interprofessional teamwork on outcome criteria on the client, staff and organization level: On the client/patient level, high quality teamwork is linked with higher satisfaction and treatment acceptance [51], improved quality of treatment [52], improved patient safety [53, 54] and better clinical outcomes [14, 16]. On the staff level, higher job satisfaction [28], greater well-being [55], improved mental health, better team climate and increased team efficiency [56] have been reported. On the organization level, high quality teamwork is associated with cost savings, higher workforce retention and reduced turnover [16, 57].

### Job satisfaction

Job satisfaction is a main outcome criterion in the IPO models for staff [58] and an extensively researched work attitude in organizational psychology [59]. The most widely accepted definition of job satisfaction has been formulated by Locke [60], who defined job satisfaction as “a pleasurable or positive emotional state resulting from the appraisal of one’s job or job experiences” ([60], p. 1304). It can be measured with one global item, which refers to employees' overall attitude towards their jobs. Job satisfaction is often used to operationalize team success [12, 61, 62]. However, there are also assessments that divide job satisfaction into different dimensions [63]. This is due to the impact of job satisfaction on the perception of quality of care and patient outcomes, such as length of hospital stay, medical errors and mortality [26, 52, 64], and its association with performance, motivation, absenteeism/tardiness, mental/physical health and general life satisfaction [65].

### The present study

**Fig. 1:**
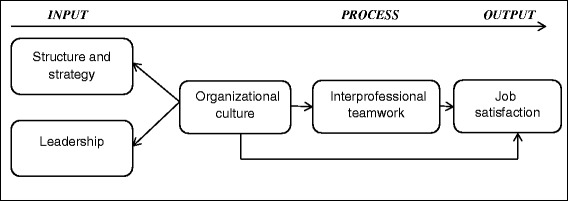
Model of the impact of organizational culture on teamwork and job satisfaction (IPO model)

Few studies have tested the mediating effects of team process variables based on the IPO model [66]. To the best of our knowledge, no study has combined organizational culture, interprofessional teamwork and job satisfaction. We developed our model (see Fig. 1) using the IPO model [11, 12, 14, 15] as a framework and considering the abovementioned research on teamwork and the impact of organizational culture on job satisfaction. Based on previous findings [30, 33–35], we also tested whether job satisfaction can be predicted through interprofessional teamwork. Moreover, we do not limit our study to one professional group, such as nurses or physicians, as practiced in most other studies, but include all kinds of different health care professionals, because interprofessional teamwork is perceived as a key feature of the comprehensive chronic care approach in rehabilitation in Germany [24, 46, 48, 67, 68].

The aim of this study was to examine the model (see Fig. 1) using structural analysis. In particular, we sought to empirically (a) assess model variables and (b) estimate the associations and predictive relationships between organizational culture, interprofessional teamwork and job satisfaction as illustrated in Fig. 1, as well as the direct association of organizational culture with job satisfaction. To this end, the following hypotheses were formulated regarding the fit of the model and the relationships within the models.

*Hypothesis 1:* The data can be adequately modeled by a theory-based structural equation model.

*Hypothesis 2*:

- Interprofessional teamwork mediates between organizational culture and job satisfaction.
- Organizational culture is also an independent predictor of job satisfaction.

## Method

This multi-center cross-sectional study was part of the project “Development and evaluation of a shared-decision-making training program in medical rehabilitation” funded by the German Federal Ministry of Research and Education and the German Statutory Pension Insurance Scheme (Grant 01GX0720). For this study, we collected data at fifteen rehabilitation clinics that treated different chronic diseases using an employee survey, which measured an individual health care professional´s perspective. Six of the centers were psychosomatic clinics and nine were somatic rehabilitation clinics with a wide range of indications (i.e., orthopedics, cardiology, neurology, oncology, metabolism and pulmonology). Each clinic designated a contact person (typically a senior physician or psychologist) who was responsible for the implementation of the study: All questionnaires (N = 662) were sent to these contact persons, who distributed them to all health care professionals at their clinic. Two weeks after the deadline, we sent out reminders to all health care professionals.

The study included all health care professionals who worked within a team and were directly involved in patient treatment. It was conducted in accordance with the Declaration of Helsinki [69]. All ethical principles for medical research involving human subjects were approved and supported by the Ethics Committee of the University of Freiburg.

### Instruments

For the assessment of *organizational culture,* a *Hospital Culture Questionnaire (HCQ)* was developed based on the Corporate Culture Scale-Short Form (CCS-SF) by Jöns [70], because there was no German questionnaire for this construct. The adaption for the clinical context takes place in a consensus process of three experts of different health care professions (psychologist, physician and physiotherapist). Since most of the original items fitted the clinical context, we only had to adapt the wording in some parts, e.g. clinic instead of company or patient instead of customer. We also omitted two items (the relationship of the staff is characterized/shaped by cooperation and the trust of the staff in their leaders is high) on the assumption that the team-orientated clinic includes this, and added a further three (conflict management, control of difficult situations, and ability to criticize in a constructive way) on the basis of a pilot study. Likewise, staff-centeredness is included as an important quality criterion for health care organizations [71]. The bipolar scaling of the CCS-SF has been simplified in a unipolar scale. This newly developed *Hospital Culture Questionnaire* consists of 14 items: six for “structure and strategy” and eight for “leadership” (see Additional file 1). The 14 items were assessed on a five-point Likert-Scale (1 = strongly agree to 5 = strongly disagree), and there was also the option of checking “no opinion”. The values of the items were recoded (1 = strongly disagree and 5 = strongly agree) before calculating the scores of the subscales *Structure and Strategy* and *Leadership* and the complete scale *Organizational culture,* because we wanted higher values to indicate a more favorable evaluation. Patient-centeredness, staff, team and quality orientation, decision-making and openness for innovations are the main topics of the items on the *Structure and strategy* subscale. A sample item is “in my view the clinic is quality-oriented.” The leadership subscale captures leadership performance, and the items focus on trust, cooperation, difficult situations, conflict management, constructive criticism, participation, information and relevance of teamwork. The complete scale *Organizational culture* consists of both subscales. The HCQ was psychometrically tested and validated [72]. Scale reliability was checked by computing Cronbach’s alpha (Structure and strategy: .85 and Leadership: .92) [72]. The two scales correlate highly (r = .52-.62) with scales which are similar in content (e.g. leadership, organization and communication and organizational climate).

*Teamwork* was measured using the *Internal Participation Scale (IPS)* [49] which captures the core dimensions of teamwork identified by Valentine, Nembhard and Edmondson [73]. The items capture communication (“*Communication in the team is efficient”*) and cooperation among health care professionals (“*The health care professionals work hand-in-hand”*), coordination of treatment options within the interprofessional team (*“The different types of treatment are well coordinated”*), coordination of the health care professionals (“*Agreements among health care professionals are well coordinated”),* respect among the health care professionals (“*The health care professionals respect each other”*) and climate within the interprofessional team (“*Overall there is a friendly climate in the clinic”*). The six IPS items are assessed on a four-point Likert scale (1 = does not apply at all, 2 = generally does not apply, 3 = generally applies, 4 = fully applies), with the additional option of responding “I cannot judge this.” When calculating the total score (team score), one missing item is accepted. The raw scores are transformed to a range from 0 (minimum level of teamwork) to 100 (highest level of teamwork). The IPS possesses good psychometric properties [49].

*Job satisfaction* was measured using the item “How satisfied are you in general with your job?” from the *Questionnaire on Staff Satisfaction in Medical Rehabilitation* by Farin et al. [74], which is assessed on a five-point Likert scale (1 = very dissatisfied, 2 = dissatisfied, 3 = neither satisfied nor dissatisfied, 4 = satisfied, 5 = very satisfied). The psychometric criteria of this item were proved to be good.

### Statistical analysis

Initially, the items were checked for plausibility, and missing data analysis was performed. Descriptive statistics were calculated using SPSS version 20.0. Structural equation modeling (SEM) was carried out using AMOS software version 20.0 (maximum-likelihood method). Data was imputed by means of the expectation-maximum algorithm to avoid biases, even in cases of missing at random (MAR) [75]. Structural equation modeling (SEM) was conducted to verify whether the proposed model reproduced the data. Several global fit measures were considered. Chi-square (*X*^2^), the Comparative Fit Index (CFI), the Tucker-Lewis Index (TLI) and the Root Mean Square Error of Approximation (RMSEA) were used as goodness-of-fit indicators. The *X*^2^-test was used as the strictest form of model testing [76]. In addition, the TLI and the CFI were calculated as measures of incremental model fit. For these measures, values ≥ .90 are suggested as criteria for an acceptable model fit and ≥ .95 for a good fit [77, 78]. The RMSEA indicates the proportion of variance-covariance information not correctly predicted by the model. Values of ≤ .08 or ≤ .05 are deemed to indicate an acceptable or good fit, respectively [77]. Furthermore, indicators of local fit were applied. The proportion of variance of the indicators (IR) predicted by the construct is supposed to amount to > .40, and the average proportion of variance (AVE) measured by the construct is supposed to be > .50 [76]. As criterion for factor reliability (FR), values > .60 are accepted as satisfactory [79]. The discriminant validity was checked using the Fornell-Larcker criterion, which requires the construct to be more strongly related to its own indicators than to other model constructs [78].

### Participants

Of 661 questionnaires sent out to members of the health care teams in the medical rehabilitation clinics, 275 were returned (41.6 %). Three questionnaires (staff members) were excluded because too much information was missing (more than 30 %), yielding a total of 272 employees that could be analyzed. Most of the items have zero to four responses with missing data (n = 15 items). Only five items have seven and more, with the maximum number at 17 (6.3 %) for the IPS item “The different types of treatment are well coordinated”.

Table 1 displays the sample characteristics. About one fourth of the participants were psychosocial therapists, and about 18 % physicians, nursing staff and physical therapists (physiotherapists and sport teachers). Most of the health care professionals were aged between 26 and 55, worked full time and had worked for more than five years at their clinic.

**Table 1:** Description of sample (n = 272)

	Frequency	Percent
Gender		
Male	94	34.6
Female	164	60.3
Missing	14	5.1
Age Groups		
17-25	12	4.4
26-35	40	14.7
36-45	82	30.1
46-55	88	32.4
56-65	38	14.0
Missing	12	4.4
Professionals		
Physicians	49	18.0
Nursing staff	48	17.7
Psychosocial therapists	67	24.6
Physical therapists	50	18.4
Others	37	13.6
More than one professional group	12	4.4
Missing	9	3.3
Job tenure		
More than one year, but less than three years	37	13.6
Three to five years	26	9.6
More than five years	190	69.9
Less than one year	13	4.8
Missing	6	2.2
Employment		
Full-time	174	64.0
Part-time (more than 70 % but less than 100 %)	41	18.0
Part-time (more than 30 % but less than 70 %)	35	15.1
Missing	14	2.9

## Results

The descriptive statistics pertaining to all scales are summarized in Table 2. The mean values (M) for all scales were neutral to positive (M ≥ 3), and standard deviations (SD) ranged between .53 and .87. Although there are significant violations of symmetry for the three scales *Structure and strategy*, *Teamwork* and *Job satisfaction*, the absolute skewness (values below 3) can be considered acceptable [76, 80]. Specifically, these three scales are left-skewed (see t-value in Table 2), but without significant ceiling effects. According to Kline [81], absolute skewness values below 3 indicate no critical violation of the normal distribution assumption.

**Table 2:** Descriptive statistics for all variables of the model

Factor	Scale range	M	SD	Skewness	t
Structure and strategy	1-5	3.39	.82	-.37	−2.46
Leadership	1-5	3.10	.87	-.07	−0.43
Organizational culture	1-5	3.23	.81	-.20	−1.33
Interprofessional teamwork	1-4	2.95	.53	-.63	−4.52
Job satisfaction	1-5	3.91	.83	-.10	−6.74

Higher values indicate a more favorable rating

The two subscales *Structure and strategy* and *Leadership* link highly with one another, and as expected, they have a high association with the complete scale *Organizational culture*. There are also high correlations between *Teamwork* and *Organizational culture* and its two subscales. The associations of all these scales with *Job satisfaction* are moderate (see Table 3).

**Table 3:** Product–moment correlations among all scales and subscales

Factor	Structure and strategy	Leadership	Organizational culture	Interprofessional teamwork	Job satisfaction
Structure and strategy	1	.78**	.92**	.59**	.44**
Leadership		1	.96**	.65**	.43**
Organizational culture			1	.66**	.46**
Interprofessional teamwork				1	.44**
Job satisfaction					1

** p < .01

### Structural equation analysis

A three-factor measurement model (*Structure and strategy, Leadership* and *Teamwork*) with a total of 20 items was used as a basis to confirm the model. The global model fit indices (see Table 4) illustrate that this original confirmatory model (model 1) explained the data acceptably (*X*^2^/df > 2, TLI <. 95, CFI < .95 and RMSEA < .05). The items *patient oriented* and *agreements* have indicator reliabilities lower than .4 (see Table 4), but were not eliminated to maintain the complete scale in the analysis. Furthermore, the substantial error correlations *(covariance modification indices (M.I.))* indicate local dependency between the items *participatory leadership style* and *trust in employees (M.I.: 30.19)*, between *cooperation* and *agreements (M.I.: 21.18)* as well as between *respect* and *communication (M.I.: 27.14),* and they were taken into account in a modified confirmatory model (model 2). With model 2, an acceptable to good fit was achieved for all fit indices (*X*^2^/df = 2, TLI = .94, CFI = .95 and RMSEA < .06). The local fit indices for model 2 are summarized in Table 5. The required thresholds for factor reliability in structural equation models (≥ .6) and average variance explained (≥ .5) have been exceeded for all scales. The threshold for indicator reliability was exceeded in 18 out of 20 items. The t-values of all factor loadings were significant.

**Table 4:** Global model fit indices for all estimated models

	*X* ^2^	df	p	*X* ^2^/df	TLI	CFI	RMSEA []
Threshold for acceptable fit	-	-	< .05	≤2.5	≥. 90	≥. 90	≤. 08
good fit	-	-	-	≤2.0	≥. 95	≥. 95	≤. 05
Confirmatory models	-	-	-	-	-	-	-
Model 1 (original confirmatory model)	406.08	167	< .001	2.43	.914	.924	[.064 .073 .082]
Model 2 (modified confirmatory model)	324.24	164	< .001	1.98	.941	.949	[.050 .060 .070]
Predictive models	-	-	-	-	-	-	-
Model 3 (IPO model)	370.80	184	< .001	2.02	.934	.943	[.052 .061 .070]
Model 4 (IO model)	156.06	88	< .001	1.72	.967	.972	[.037 .052 .065]

*I,* Input (organizational culture)

*P,* Process (interprofessional teamwork)

*O,* Output (job satisfaction)

**Table 5:** Measures of local fit for modified confirmatory model

Scales	Items	IR	CR	FR	AVE
Threshold for acceptable fit		≥ .4	|C.R.| > 2, p < .05	≥ .6	≥ .5
Structure and strategy	1 Patient oriented	.34	-		
	2 Staff oriented	.66	9.83***		
	3 Quality oriented	.51	9.10***	.85	.50
	4 Open for innovations	.48	8.93***		
	5 Team oriented	.66	9.82***		
	6 Quick decision-making	.29	7.46***		
Leadership	7 Participatory leadership style	.62	13.73***		
	8 Trust in employees	.46	12.34***		
	9 Valuing internal communication	.59	13.38***		
	10 Staff participation	.65	14.19***		
	11 Open conflict management	.54	12.70***	.92	.58
	12 Appreciation of interprofessional teamwork	.53	12.52***		
	13 Managing difficult situations with employees	.59	-		
	14 Constructive criticism	.65	14.16***		
Interprofessional teamwork	15 Climate	.62	-		
	16 Cooperation	.58	12.34***		
	17 Coordination	.47	10.89***	.85	.50
	18 Agreements	.29	8.57***		
	19 Respect	.50	11.35**		
	20 Communication	.51	11.58***		

*IR,* Indicator reliability; *CR,* Critical ratio; *FR*, Factor reliability; *AVE*, Average variance extracted

***p < .001, **p < .01

**Fig. 2:**
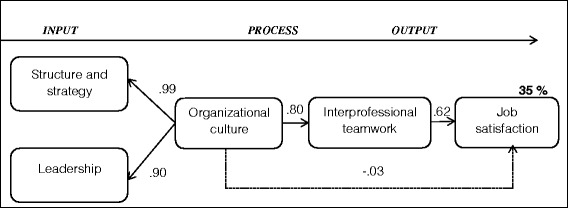
Structural equation IPO model for the prediction of job satisfaction (Model 3). The figures on the paths are the standardized path coefficients. To ensure identifiability, the indicator paths *leadership* and *structure and strategy* were fixed to 1

**Fig. 3:**
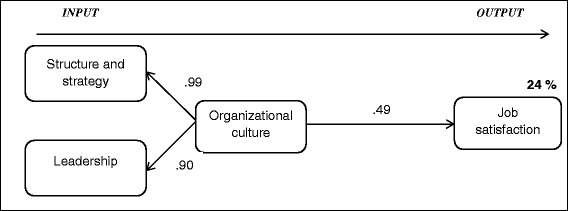
Structural equation IO model for the prediction of job satisfaction (Model 4). The figures on the paths are the standardized path coefficients. To ensure identifiability, the indicator paths *structure and strategy* and *leadership* were restricted (*leadership* = 1 and *structure and strategy* = .7)

The high correlation between the two latent factors *structure and strategy* and *leadership* (.90) support the factor of a second-order *organizational culture* as theoretically assumed. Next, with the predictive models we took the modified confirmatory model (model 2) as the base and added this second-order factor as well as *job satisfaction* as criteria (model 3: IPO and model 4: IO). Good model fit proved to be stable for these prediction models (see Table 4). The first predictive model (model 3: IPO) has a better fit than model 2 (confirmatory model with second-order factor), rendering the global fit acceptable to good. Model 3 predicts 35 % of *job satisfaction* (see Fig. 2). Thereby - as expected - the association between each of the two subscales, *structure and strategy* and *leadership,* and the complete scale, *organizational culture*, is significant (β_leadership_ = .99 and β_structure and strategy_ = .80, p < .001). *Interprofessional teamwork* is a significant predictor of *job satisfaction* (standardized regression weight: β = .80; p < .001), but *organizational culture* is not (β = −.033; p = .57). Next, when removing *interprofessional teamwork* with its six items from the IPO model, *organizational culture* becomes a significant predictor of *job satisfaction* (β = .47, p < .001). The fit of the IO model improved compared to model 3 (*X*^2^/df = 1.72 TLI = 97, CFI = .97 and RMSEA = .5). However, the explained variance in *job satisfaction* decreased to 24 % (R^2^) (see Table 4 and Fig. 3). All latent factors can be delimited from one another to a sufficient degree, as the correlations are always lower than the square root of average variance extracted (Fornell–Larcker criterion, [78, 82]).

## Discussion

Based on the IPO model [11, 12, 14, 15] and empirical findings on the influence of organizational culture [30, 33, 34] and interprofessional teamwork [27, 28] on job satisfaction, a theoretical model was developed that defines organizational culture as an input variable, interprofessional teamwork as a process variable and job satisfaction as the output variable. The confirmatory models (model 1 and model 2) were supported by the data (Hypothesis 1). Furthermore, the results showed that 35 % of job satisfaction is predicted by a structural equation model that includes both *organizational culture* and *Interprofessional teamwork* (Hypothesis 2a). The comparison of model 3 (*IPO: organizational culture – interprofessional teamwork – job satisfaction)* and model 4 *(IO: organizational culture – job satisfaction*) showed that the effect of organizational culture is completely mediated by interprofessional teamwork. The global fit indices are a little better for model 4 (TLI: .967, CFI: .972, RMSEA .052) than for model 3 (TLI: .934, CFI: .943, RMSEA: .61), but the prediction of job satisfaction is better in the IPO model (model 3) (R^2^ = 35 %) than in the IO model (model 4) (R^2^ = 24 %). The IO model supports Hypothesis 2b, which postulated that organizational culture predicts job satisfaction independently [30, 33–35]. However, if we include interprofessional teamwork (model 3), organizational culture loses its predictive value for job satisfaction, and interprofessional teamwork mediates the relationship between these two constructs completely. Few studies have explicitly investigated the mediator effect of teamwork based on the IPO model [83–85], and they were not performed in the health care setting. Most of the health care studies show the single influences of organizational culture on teamwork [23, 24, 32] or teamwork on job satisfaction [25–28] or organizational culture on job satisfaction [30, 33, 35], but there is no comparable study in health care which considers all three constructs together. Tsai [30] showed the relationships between organizational culture, leadership behavior and job satisfaction. We used leadership as a dimension of organizational culture and not as a separate construct. We also retrieved a high correlation of the constructs in our study and solved this by using “organizational culture” as a higher level factor. Leadership style is important for organizational culture as well as for interprofessional teamwork [45]. Schein [86] emphasized that culture and leadership style are “two sides of the same coin” [p. 28], since executives create culture and are influenced by it. Further findings [30, 33, 87] to the association of organizational culture and job satisfaction are also consistent with our IO model.

Furthermore Chang et al. [26] showed that interprofessional teamwork was one of the most important predictors of job satisfaction as stated in the IPO models [14, 88]. Taking the IPO model [11, 12, 14, 15] as a framework and using organizational culture as an input variable, we find a complete mediation of the effect of organizational culture on job satisfaction through teamwork. In other words, organizational culture affects job satisfaction presumably through the intermediate process - interprofessional teamwork. Thus it could be assumed that it is most important to establish good interprofessional teamwork in order to increase job satisfaction in health care. In line with previous study findings [28, 52, 88–90], this stresses the importance of high quality interprofessional teamwork in health care. From a practical point of view, these findings provide essential clues for enhancing interprofessional teamwork and ensuring high job satisfaction. There are several interventions available for improving interprofessional teamwork [12, 52, 91, 92], but there are only a few studies on this issue [93].

A limitation of this finding is the overlapping of the constructs of interprofessional teamwork and organizational culture. Theoretically, the constructs cannot be differentiated precisely; for example, teamwork is used as a dimension of organizational culture [45], or leadership (in our study, a scale of organizational culture) is also a dimension of teamwork and climate (in this study, an item of teamwork, IPS) is highly associated with culture [70]. Empirically, there is also a high correlation between organizational culture and teamwork (r = .66). Strasser et al. [32] also find associations between types of organizational culture and teamwork. The highest team functioning scores were found in teams with a dynamic culture and the lowest scores in a formalized and structured culture. Organizational culture can support teamwork [32, 94, 95]. This leads us to the conclusion that both aspects – interprofessional teamwork and organizational culture – are equally important for job satisfaction. Concerning organizational culture, the leadership is supposed to be the most important characteristic [40, 45]. “It was found that encouragement and support by leaders, their trust and clear vision, their consistent behavior in this regard and their ability to convince subordinates to acknowledge their vision can all influence employee job satisfaction.” ([30], p. 105).

The problem of overlapping can also be found in the conceptualization of IPO models, where team aspects often cannot be clearly categorized as input or process (e.g. in some models leadership is used as an input variable and in others as a process variable). It can be assumed that there are interaction effects between input and process variables [96]. In our case, the IPO models only consider a linear path from organizational culture to interprofessional teamwork, but there could also be feedback loops or effects the other way around [97]. For example, Aydin and Ceylan stated “that employee satisfaction and customer orientation has a mid-level effect on creating a substantial organizational culture” ([98], p. 1203).

Moreover, studies in health care often only focus on nurses [30] or physicians [97] and not on the interprofessional team. The health care professionals often differ in their culture, which is a barrier for effective interprofessional teamwork [99–104]. Breaking down health care professional silos (see e.g. [8, 103]) or rather working on a common culture helps to improve interprofessional teamwork as well as job satisfaction. Longitudinal intervention studies are needed to prove this assumption.

In order to consider German culture, we did not translate an existing instrument, but took an existing German instrument (the Corporate Culture Scale - Short Form (CCS-SF) by Jöns et al. [70]), adapted it to the interprofessional clinical context and validated it [72]. Although a range of instruments are globally accessible, to the best of our knowledge the HCQ is the first questionnaire specifically developed for measuring organizational or rather clinical culture in an interprofessional health care context in Germany. Instruments in health care often focus on selected aspects such as safety culture (e.g. [105]). HCQ focuses thereby on the main quality aspects, such as patient-, staff-, and quality-centeredness of clinics. Scott et al. [45] stated that there is no ideal instrument; they all have their limitations.

The study results confirm our recommendation to focus more on organizational aspects of the clinics such as structure, strategy and leadership in order to improve effectiveness and efficiency. It is known from several studies that good interprofessional teamwork and job satisfaction are essential preconditions for high quality care and clinical effectiveness [26, 35, 106, 107]. Ramanujam and Rousseau summarized this very appropriately: “The challenges are organizational and not just clinical” ([107]; p. 811) and described that thinking “organizationally has powerful implications for health care organizations” [p. 823], because the staff and the teams are embedded in an organization which impacts their behavior, the teamwork, etc. [94].

### Limitations and strengths

The study has methodological limitations. The generalizability of the results for all in-patient medical rehabilitation clinics in Germany is limited because we only covered 15 clinics in southwest Germany, and the questionnaire return rate was below 50 %, which was stated as acceptable by Bungard and Jöns [108]. Yet, our rate of 42 % is fairly good in comparison with other studies in health care, where the average return rate for employee surveys was between 30 and 50 % [28, 109]. Due to the voluntary participation of the clinics as well as the clinic staff, selection effects are likely to have occurred. It can be assumed that motivated clinics and staff were more likely to participate. The representativeness of our sample remains an open issue, as we did not obtain background data about the staff in the clinics (e.g., team composition, gender and age).

The medium to high correlations of the constructs could be the result of overlapping constructs [63, 70, 110] and of common-method bias [111]. We also rely on self-reported data, potentially giving rise to single-source bias. Furthermore, the study’s design is cross-sectional and hence does not allow for a causal interpretation of the relationships found in the predictive model. Longitudinal or intervention studies should be conducted to examine the causality of the proposed relationships. Despite the low indicator reliability (≤.4) [79] of three items (see Table 5: items ‘patient-oriented’, ‘quick decision making’ and ‘agreements’), we did not eliminate them from the model in the SEM because we wanted to retain the contents.

However, the study also possesses some specific strengths. It is a multi-center study, and it confirmed with a good model fit, a theory-based model designed on the basis of the IPO model. To our knowledge, no other study has been published on this issue in an interprofessional health care context. In general, few studies focus on organizational factors in health care organizations. Germany in particular is lacking in this field. The study therefore delivers results in a research field where empirical findings are rather rare [66]. The mediation of the effect of organizational behavior on job satisfaction through interprofessional teamwork in health care organizations has not been tested before. Further studies should investigate the constructs and their interdependence in a more differentiate manner.

## Conclusions

With regard to practical implications of the results, managers of health care organizations are advised to increase their awareness concerning the importance of good organizational culture and interprofessional teamwork. Furthermore, the implementation of team development interventions can be recommended and should be supported. Any such intervention should be tailored to the needs of the teams, and further research should evaluate these interventions to find evidence-based best practices. The evaluation of these interventions should rely on a set of different assessment methods. It is desirable to put more weight on behavior, which might be assessed through observation and it would be interesting to consider additional economic and outcome parameters on the patient side (e.g., treatment success and patient satisfaction). In order to combine organizational, team and patient data, the multi-level-approaches are the state of the art. Our study results underpin the importance of investigating the characteristics and effects of working conditions in health care facilities more comprehensively. The study is a foundation for further longitudinal intervention studies and multi-level approaches.

## Additional file

Additional file 1:
**The hospital culture questionnaire.**

## Abbreviations

AVE: Average variance extracted
CFI: Comparative Fit Index
CR: Critical ratio
df: Degree of freedom
FR: Factor reliability
IPS: Internal Participation Scale
IR: Indicator reliability
MAR: Missing at random
M.I.: Modification indices
M: Mean value
RMSEA: Root Mean Square Error of Approximation
SD: Standard deviations
SEM: Structural equation modeling
TLI: Tucker-Lewis index
*X*^2^: Chi-Squared value
*X*^2^/df: Normed Chi-Squared value
