# Supplementary material for: Relationship of organizational culture, teamwork and job satisfaction in interprofessional teams
Source: BMC Health Serv Res. 2015 Jun 23;15:243. doi: 10.1186/s12913-015-0888-y (PMC4477418; doi:10.1186/s12913-015-0888-y)
Supplement: Additional file 1: — The hospital culture questionnaire. [file 12913_2015_888_MOESM1_ESM.docx]

**The *Hospital Culture Questionnaire* (HCQ)**

(based on the Corporate Culture Scale - Short Form by Jöns et al., 2005)

How do you personally perceive the clinic and managers concerning the following aspects?

| **In my view the clinic is...** | strongly agree | agree  somewhat | un-decided | disagree  somewhat | strongly disagree | no opinion |
| --- | --- | --- | --- | --- | --- | --- |
| 1. …patient-centred | ➀ | ➁ | ➂ | ➃ | ➄ | O |
| 1. …staff-centred | ➀ | ➁ | ➂ | ➃ | ➄ | O |
| 1. …quality-orientated | ➀ | ➁ | ➂ | ➃ | ➄ | O |
| 1. …open for innovation | ➀ | ➁ | ➂ | ➃ | ➄ | O |
| 1. ...team-orientated | ➀ | ➁ | ➂ | ➃ | ➄ | O |
| 1. ...enjoys direct and efficient decision-making channels | ➀ | ➁ | ➂ | ➃ | ➄ | O |

| **The clinic managers…** | strongly agree | agree  somewhat | un-decided | disagree somewhat | strongly disagree | no opinion |
| --- | --- | --- | --- | --- | --- | --- |
| 1. ...demonstrate a participative leadership style | ➀ | ➁ | ➂ | ➃ | ➄ | O |
| 1. ...place a high level of trust in their staff | ➀ | ➁ | ➂ | ➃ | ➄ | O |
| 1. ...attach great importance to internal communication (staff information) | ➀ | ➁ | ➂ | ➃ | ➄ | O |
| 1. ...involve staff in decisions | ➀ | ➁ | ➂ | ➃ | ➄ | O |
| 1. ...address conflicts openly | ➀ | ➁ | ➂ | ➃ | ➄ | O |
| 1. ...attach great importance to interdisciplinary teamwork | ➀ | ➁ | ➂ | ➃ | ➄ | O |
| 1. ...have difficult situations with their staff under control | ➀ | ➁ | ➂ | ➃ | ➄ | O |
| 1. ...are able to express criticism in a constructive way | ➀ | ➁ | ➂ | ➃ | ➄ | O |
